# Supplementary material for: The interactome of intact mitochondria by cross-linking mass spectrometry provides evidence for coexisting respiratory supercomplexes
Source: Mol Cell Proteomics. 2017 Dec 8;17(2):216–32. doi: 10.1074/mcp.RA117.000470 (PMC5795388; doi:10.1074/mcp.RA117.000470)
Supplement: Supplemental Data [file supp_RA117.000470_133922_0_supp_23978_fzffwf.zip › spectra_annotation/mito_DR_spectra_annotation/213-1-2-1-1-1.pdf]

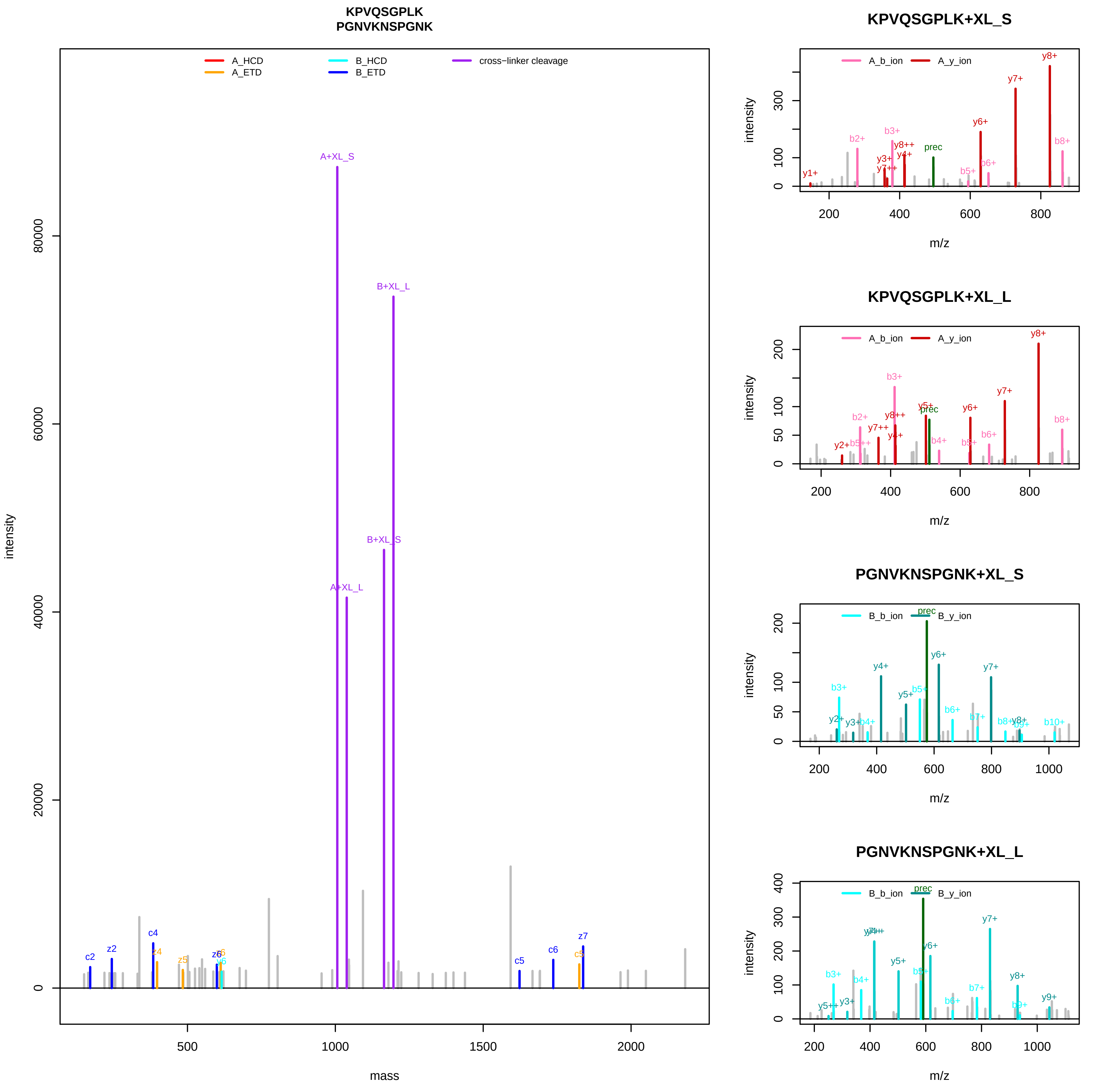

KPVQSGPLK
PGNVKNSPGNK
A_HCD
A_ETD
B_HCD
B_ETD
cross−linker cleavage
A+XL_S
B+XL_L
B+XL_S
A+XL_L
c2
z2
c4
z4
z5
z6
c6
y6
c5
c6
c5
z7
intensity
mass
0
20000
40000
60000
80000
500
1000
1500
2000
KPVQSGPLK+XL_S
A_b_ion
A_y_ion
y1+
b2+
y3+
y7++
b3+
y8++
y4+
prec
b5+
b6+
y6+
y7+
y8+
b8+
intensity
m/z
KPVQSGPLK+XL_L
A_b_ion
A_y_ion
y2+
b2+
b5++
y7++
b3+
y8++
y4+
y5+
prec
b4+
b5+
y6+
b6+
y7+
y8+
b8+
intensity
m/z
PGNVKNSPGNK+XL_S
B_b_ion
B_y_ion
prec
y2+
b3+
y3+
b4+
y4+
y5+
b5+
y6+
b6+
b7+
y7+
b8+
y8+
b9+
b10+
intensity
m/z
PGNVKNSPGNK+XL_L
B_b_ion
B_y_ion
prec
y5++
b3+
y3+
b4+
y4++
y5+
b5+
y6+
b6+
b7+
y7+
y8+
b8+
y9+
intensity
m/z
